# Whole-Genome Metagenomic Analysis of the Gut Microbiome in HIV-1-Infected Individuals on Antiretroviral Therapy

**DOI:** 10.3389/fmicb.2021.667718

**Published:** 2021-06-25

**Authors:** Xiangning Bai, Aswathy Narayanan, Piotr Nowak, Shilpa Ray, Ujjwal Neogi, Anders Sönnerborg

**Affiliations:** ^1^Division of Clinical Microbiology, Department of Laboratory Medicine, ANA Futura, Karolinska Institutet, Stockholm, Sweden; ^2^Division of Infectious Diseases, Department of Medicine Huddinge, Karolinska Institutet, Stockholm, Sweden; ^3^State Key Laboratory of Infectious Disease Prevention and Control, National Institute for Communicable Disease Control and Prevention, Chinese Center for Disease Control and Prevention, Beijing, China; ^4^Division of Laboratory Medicine, Oslo University Hospital, Oslo, Norway; ^5^Department of Infectious Diseases, Karolinska University Hospital, Stockholm, Sweden; ^6^The Laboratory for Molecular Infection Medicine Sweden MIMS, Umeå University, Umeå, Sweden

**Keywords:** gut microbiome, shotgun metagenome sequencing, HIV-1 infection, virulome, resistome

## Abstract

Gut microbiome plays a significant role in HIV-1 immunopathogenesis and HIV-1-associated complications. Previous studies have mostly been based on 16S rRNA gene sequencing, which is limited in taxonomic resolution at the genus level and inferred functionality. Herein, we performed a deep shotgun metagenomics study with the aim to obtain a more precise landscape of gut microbiome dysbiosis in HIV-1 infection. A reduced tendency of alpha diversity and significantly higher beta diversity were found in HIV-1-infected individuals on antiretroviral therapy (ART) compared to HIV-1-negative controls. Several species, such as *Streptococcus anginosus*, *Actinomyces odontolyticus*, and *Rothia mucilaginosa*, were significantly enriched in the HIV-1-ART group. Correlations were observed between the degree of immunodeficiency and gut microbiome in terms of microbiota composition and metabolic pathways. Furthermore, microbial shift in HIV-1-infected individuals was found to be associated with changes in microbial virulome and resistome. From the perspective of methodological evaluations, our study showed that different DNA extraction protocols significantly affect the genomic DNA quantity and quality. Moreover, whole metagenome sequencing depth affects critically the recovery of microbial genes, including virulome and resistome, while less than 5 million reads per sample is sufficient for taxonomy profiling in human fecal metagenomic samples. These findings advance our understanding of human gut microbiome and their potential associations with HIV-1 infection. The methodological assessment assists in future study design to accurately assess human gut microbiome.

## Introduction

It is well-recognized that gut microbiome (GM) is a key player in intestinal and physiological homeostasis, immunity, and energy metabolism. Dysbiosis in the GM may alter intestinal barrier functions, host metabolic and signaling pathways, which are directly or indirectly linked to various diseases and metabolic disorders such as obesity (Turnbaugh et al., 2006), diabetes (Sonnenburg and Backhed, 2016), autoimmune diseases (Severance et al., 2016), cardiovascular disease (Karlsson et al., 2013), inflammatory bowel disease (Ananthakrishnan et al., 2018), cancer (Schwabe and Jobin, 2013), and neuropsychiatric disorders (Mathee et al., 2020). Accumulating studies in recent years have indicated that GM plays a significant role in human immunodeficiency virus type 1 (HIV-1) immunopathogenesis as well as in HIV-1-associated chronic complications (Vujkovic-Cvijin et al., 2013; Nowak et al., 2015; Dillon et al., 2016). The gastrointestinal tract is severely and rapidly damaged following HIV-1 infection (Hunt et al., 2014), resulting in structural impairment of the epithelial barrier and a disruption of intestinal homeostasis. The resulting translocation of bacterial products and bacteria themselves from the lumen into the systemic circulation is associated with local and systemic inflammation, immune activation, and deregulation (Svard et al., 2015), which are not fully restored with antiretroviral therapy (ART) (Dillon et al., 2016; Ray et al., 2020).

Cross-sectional studies have shown reduced gut microbiota diversity and compositional shifts from *Bacteroides* to *Prevotella* predominance after HIV-1 infection (Vazquez-Castellanos et al., 2015; Ling et al., 2016; Dillon et al., 2017; Lu et al., 2018), which have been linked to lower CD4 + T-cell count, higher inflammation, and increased immune activation (Vujkovic-Cvijin et al., 2013; Dinh et al., 2015). However, in some studies, such microbiota shifts have been observed neither in animal models (Handley et al., 2012) nor in studies matching for HIV-1 risk groups (Noguera-Julian et al., 2016; Kelley et al., 2017). Importantly, previous microbiome studies in HIV-1 infection have mostly been based on 16S rRNA gene sequencing approach, which has several limitations, for instance, taxonomic resolution at the genus level (Knight et al., 2018). To date, limited HIV-1 microbiome studies have been conducted using whole metagenome sequencing, which can characterize the microbiome at the species and gene levels. The sequencing depth of a few published studies was inadequate (Vazquez-Castellanos et al., 2015, 2018; D’Souza et al., 2019; Rocafort et al., 2019) and underpowered to identify granular differences in both microbial composition and function (Rocafort et al., 2019). It is noteworthy that one previous study indicated that enrichment of bacterial virulence factors and antimicrobial resistance (AMR) genes were observed in HIV-1-infected individuals with progressively lower nadir CD4 + T-cell counts (Guillen et al., 2019). It remains to be seen if such alterations contribute to immune suppression in HIV-1 infection. A reliable and comprehensive pattern of gut microbiome dysbiosis and its impact on HIV-1 disease progression are yet to be fully deciphered.

In the current study, we set up whole metagenomics workflows and characterized the gut microbiome features in terms of microbial composition at the species level, functional genes, metabolic pathways, bacterial virulence factors, and AMR genes, with respect to HIV-1 infection. As previous studies have shown that different DNA extraction methods have an impact on DNA yield and quality, which may influence the structure of bacterial communities (Costea et al., 2017; Angebault et al., 2018; Lim et al., 2018), we thus firstly evaluated the effect of different genomic DNA (gDNA) extraction protocols on the DNA quantity and quality. Whole metagenome sequencing was then performed on fecal samples from HIV-1-infected individuals on ART (HIV-1-ART) from the Swedish InfCareHIV cohort, as well as HIV-1-negative controls. We evaluated whole metagenome sequencing depth required for recovery of the bacterial species, virulence factors genes, and AMR genes. We assessed proportions of different types of microbes and functionally characterized genes in human gut metagenomic samples. Finally, metagenomics data were analyzed in combination with clinical variables to determine potential associations between gut microbiome and HIV-1 infection.

## Materials and Methods

### Study Participants

This cross-sectional study included HIV-1-ART patients who were recruited from the outpatient clinic at Karolinska University Hospital, Stockholm, Sweden, and HIV-1-negative controls. The inclusion criteria for the HIV-1-ART participants were age >18 years (median: 45 years; range: 38–62 years), and HIV-1 positive for at least 6 months. The exclusion criteria were ongoing HIV-1-related complications or antibiotic treatment during the previous 3 months. The patients received ART for a median of 7.7 years (range: 4.4–20.8 years). The HIV-1-negative controls were healthy individuals (median: 32 years; range: 24–51 years) who did not receive antibiotic treatment during the last 3 months.

This study was approved by the Regional Ethics Committee, Stockholm (2009/1485-31, 2013/1944-31/4, 2014/920-3).

### Samples and Data Collection

A sterile tube for fecal sampling without preservation media was used when participants were able to donate feces at the clinic as previously described (Vesterbacka et al., 2017). Samples were frozen and stored at −80°C within 24 h. Stool collection tube with DNA stabilizer (Stratec Biomedical) was used for participants who collected the feces at home. The samples were delivered to the outpatient clinic by the participants or instantly sent by post and stored at −80°C according to the manufacturer’s instructions.

Clinical data of the HIV-1-ART participants were collected from the InfCareHIV database, including gender, age, current, and nadir CD4 + T-cell counts, ART regimen at sampling date, total ART duration (years), and transmission mode (Table 1).

**TABLE 1:** Characteristics of study subjects.

				**CD4 + T-cell count**				
				**(cells/mm^3^)**				
**Sample ID**	**Group**	**Age at sampling date (years)**	**Gender**	**Current**	**Nadir**	**ART at sampling date**	**Total ART duration (years)**	**Transmission mode**
Q-C1	Case	43	Male	440	270	Abacavir/Lamivudine/Dolutegravir	4.4	Heterosexual
Q-C2	Case	48	Female	400	150	Abacavir/Lamivudine/Dolutegravir	8.3	Heterosexual
Q-C3	Case	38	Male	770	344	Tenofovir (TAF)/Emtricitabine/Rilpivirine	6.4	MSM
Q-C4	Case	42	Female	810	530	TDF/Emtricitabine/Rilpivirine	5.1	Heterosexual
Q-C5	Case	43	Female	740	430	TDF/Emtricitabine/Dolutegravir	5.7	Heterosexual
Q-C6	Case	54	Female	690	280	TDF/Emtricitabine/Efavirenz	6.8	Heterosexual
Q-C7	Case	56	Female	800	570	TDF/Emtricitabine/Rilpivirine	5.0	Heterosexual
Q-C8	Case	45	Male	680	300	Abacavir/Lamivudine/Dolutegravir	8.1	Heterosexual
Q-C9	Case	62	Male	330	140	TAF/Emtricitabine/Dolutegravir	8.8	Drug use
Q-C10	Case	38	Female	780	300	Abacavir/Lamivudine/Dolutegravir	7.7	Heterosexual
Q-D2	Case	44	Female	1020	273	Dolutegravir/Abacavir/Lamivudine	20.8	Heterosexual
Q-D3	Case	55	Female	390	170	TDF/Emtricitabine/Dolutegravir	20.7	Blood product
Q-D4	Case	50	Female	970	380	TDF/Emtricitabine/Dolutegravir	10.9	Heterosexual
Q-H1	Control	29	Male	-	-	-	-	-
Q-H2	Control	35	Male	-	-	-	-	-
Q-H3	Control	51	Male	-	-	-	-	-
Q-H4	Control	32	Male	-	-	-	-	-
Q-H5	Control	40	Male	-	-	-	-	-
Q-H6	Control	24	Male	-	-	-	-	-
Q-H7	Control	30	Male	-	-	-	-	-
Q-H8	Control	27	Male	-	-	-	-	-
Q-H9	Control	42	Male	-	-	-	-	-
Q-H10	Control	33	Male	-	-	-	-	-
Q-H11	Control	30	Male	-	-	-	-	-

### Metagenomic DNA Extraction With Different Protocols

First, we did literature review and selected and evaluated two widely used protocols, i.e., QIAamp PowerFecal Pro DNA Isolation kit (QP) (Qiagen, Germany) and the standardized International Human Microbiota Standards (IHMS) Protocol Q recommended by the International Human Microbiome Consortium^1^ (Costea et al., 2017), which performed better than other methods in terms of both DNA quality and microbial diversity in human fecal microbiota studies (Angebault et al., 2018; Lim et al., 2018; Fiedorova et al., 2019), while never having been compared before. A subset of fecal samples was randomly selected from cases and controls and then subjected to the two protocols according to instructions with minor modifications. The quantity and quality of gDNA extracted by the two protocols were measured using NanoDrop^TM^ One (Thermo Scientific, United States) and Agilent 2200 TapeStation (Agilent Technologies, United States) including gDNA concentration, purity, and integrity. The protocol yielding higher gDNA quantity and better quality was then used to extract gDNA of the remaining samples. The gDNA samples were stored at −80°C until library preparation and sequencing.

### Library Preparation and Shotgun Metagenome Sequencing

Sequencing libraries were prepared with the Nextera DNA Flex kit (Illumina, Inc.) following the manufacturer’s instructions. One paired-end (PE) library with insert size of approximately 320 bp was constructed for each sample. Libraries were normalized with Qubit assay; the pooled library was then sequenced on one lane on NovaSeq6000 platform (NovaSeq Control Software 1.6.0/RTA v3.4.4) with a 2 × 150 setup using “NovaSeqXp” workflow in “S1” mode flowcell. The Bcl to FastQ conversion was performed using bcl2fastq_v2.20.0.422 from the CASAVA software suite. The quality scale used is Sanger/phred33/Illumina 1.8 +.

### Data Preprocessing

**FIGURE 1:**
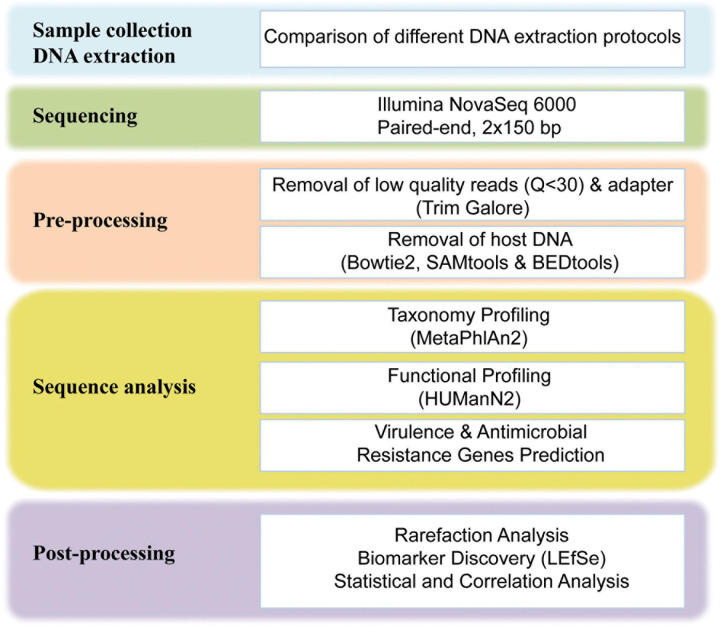
Schematic overview of the study design and workflow. For more details on each step of the workflow, see *Materials and Methods*.

A total of 146 GB raw sequencing data were generated for 24 fecal samples, approximately 6.1 GB per sample was obtained. The raw sequencing data were preprocessed using our in-house bioinformatics pipelines (Figure 1). Briefly, the adapter and low-quality reads (a quality score of less than Q30) were removed using Trim galore (v0.6.4).^2^ After the quality trimming, Bowtie2 (v2.3.5.1) (Langmead and Salzberg, 2012) was used in combination with SAMtools (v1.19) (Li et al., 2009) and BEDtools (v2.29.2) (Quinlan and Hall, 2010) to identify and remove human DNA sequences. The human unmapped reads were then used for downstream analysis. After filtering, an average of 41 million 150-bp PE reads per sample was obtained, the adapter and low-quality reads accounted for 1.32% of total reads, and the average rate of human DNA contamination in all samples was 0.58% (Supplementary Table S1).

### Taxonomical Profiling

The taxonomic assignment and abundance estimation were performed with MetaPhlAn 2.0 (Truong et al., 2015) using default parameters. MetaPhlAn 2.0 relies on ∼1 million unique clade-specific marker genes identified from ∼17,000 reference genomes (∼13,500 bacterial and archaeal, ∼3,500 viral, and ∼110 eukaryotic genomes) (Truong et al., 2015). This computational tool provides the relative abundances of each microbial clade with species-level resolution. The filtered reads were mapped to all reference genome sequences to determine the presence and abundance of different taxonomic groups present in human fecal samples. Given that this study focused on bacterial communities, downstream analysis was performed on bacterial taxa.

### Microbial Gene Richness Assessment

Filtered reads were mapped against the integrated gene catalog (IGC) (Li et al., 2014) using the bwa software (Li and Durbin, 2009). Gene richness was measured as the total number of different genes present in the sample regardless of their abundance and length. A minimum of one filtered mapped read was set to consider the presence of a gene, as described previously (Guillen et al., 2019).

### Functional Genes and Pathway Analysis

Metagenomics functional analysis was performed using the HMP Unified Metabolic Analysis Network 2 (HUMAnN2), which identifies the species profile from metagenomic shotgun sequencing data and aligns reads to their pan-genomes, performs translated search on unclassified reads, and quantifies gene families and pathways (Franzosa et al., 2018). By default, gene families were annotated using a comprehensive protein database UniRef90 (Suzek et al., 2015) and metabolic pathways using MetaCyc database (Caspi et al., 2016). The UniRef90 gene family abundance from HUMAnN2 was then regrouped to Kyoto Encyclopedia of Genes and Genomes (KEGG) (Kanehisa and Goto, 2000) orthology (KO). To gain a comprehensive profile of functional pathways, the filtered reads were further mapped to the KEGG database, which contains more compounds while having less reactions and pathways than does the MetaCyc database (Altman et al., 2013).

### Characterization of Gut Microbial Virulome and Resistome

To characterize the presence of bacterial virulence factors genes and AMR genes, we mapped filtered reads from each sample against the VFDB (Liu et al., 2019) and the CARD (Alcock et al., 2020), respectively, using Bowtie2 (v2.3.5.1). The mapped read segments were estimated using SAMtools idxstats. The copy number of each gene was estimated by dividing the total reads mapping to a gene divided by the gene’s length. The relative abundance of genes was calculated using R function make_relative in R package funrar (v1.4.1).

### Rarefaction Analysis of Microbiome, Virulome, and Resistome

Rarefaction curves were generated using R function rarecurve (R package vegan v2.5.6) to assess the saturation of samples at different sequencing depth for recovery of bacterial species, bacterial virulence factors genes, and AMR genes.

### Statistical Analysis

Species richness and Shannon diversity were calculated using R function estimate_richness. Beta diversity was measured by Bray–Curtis, weighted UniFrac, and unweighted UniFrac distances using R package Phyloseq (v1.30.0) (McMurdie and Holmes, 2013). Samples were clustered according to their species composition using non-metric multidimensional scaling (NMDS) approach based on Bray–Curtis, weighted UniFrac, and unweighted UniFrac distances in Phyloseq (v1.30.0).

Due to the small sample size in this study, different methods were used to determine and verify the results obtained from the other method. Differences in abundance of bacterial species, pathways, bacterial virulence genes, and AMR genes between two groups were determined using Wilcoxon rank sum test with a significance level of <0.05. The linear discriminant analysis (LDA) effect size (LEfSe) algorithm (Segata et al., 2011) was used to identify specific bacterial taxa and metabolic pathways as taxonomic and functional biomarkers and to verify the Wilcoxon rank sum test. Kruskal–Wallis test was used to process the dataset with LEfSe alpha values set at 0.05. The threshold used to consider a discriminative feature for the logarithmic LDA score was set at >2. The biomarker discovery was performed at bacterial order, family, genus, and species levels and all functional levels. All the correlation analyses were performed using the Spearman’s rank correlation coefficient (library “psych”; function “corr.test”). Spearman’s correlation (rho) with *p* < 0.05 was considered statistically significant. Statistical correction for multiple testing was not applied because of the low sample size and the exploratory focus of this study.

## Results

### Study Subjects

The study subjects were categorized into case (HIV-1-ART individuals, *n* = 13) and control (HIV-1-negative controls, *n* = 11) groups. Cases were classified into groups based on median value (740 cells/mm^3^) of current CD4 + T-cell counts: ≥740 cells/mm^3^ (*n* = 7) and <740 cells/mm^3^) (*n* = 6).

### Influence of Extraction Protocols on Genomic DNA Quantity and Quality

**FIGURE 2:**
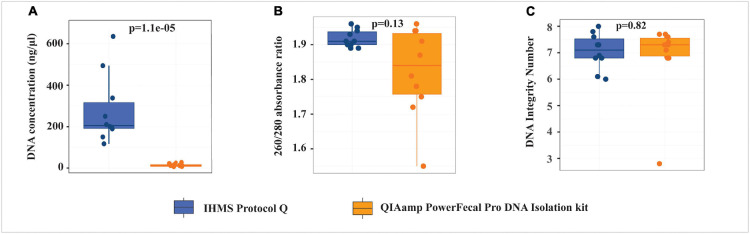
Comparison of the quantity and quality of genomic DNA extracted from QIAamp PowerFecal Pro DNA Isolation kit (QP) and IHMS Protocol Q. **(A)** DNA concentration. **(B)** 260/280 absorbance ratio. **(C)** DNA Integrity Number.

Fecal samples were randomly selected from cases (*n* = 3) and controls (*n* = 7) to evaluate two gDNA extraction protocols. Using the same fecal material mass, IHMS Protocol Q produced significantly higher gDNA yield (*p* < 0.0001, median DNA concentration 17.26 times higher) than the QP kit (Figure 2 and Supplementary Table S2). The 260/280 absorbance ratio was used to assess the gDNA purity, within an acceptable range of 1.8–2.0 as good-quality DNA. gDNA from the IHMS Protocol Q had 260/280 ratio ranging from 1.89 to 1.96, whereas four out of 10 samples extracted from the QP kit had 260/280 ratio below 1.8. The gDNA integrity was assessed with DNA Integrity Number (DIN) ranging from 1 to 10, where 1 indicates highly degraded gDNA and 10 represents highly intact gDNA (Nguyet et al., 2014). No significant difference in DIN values was found between the two protocols (*p* = 0.82); however, one sample with lower 260/280 ratio of 1.55 from the QP kit had also appreciably low gDIN value of 2.8. Our study indicated that the IHMS Protocol Q yielded significantly higher gDNA quantity and more stable gDNA quality than the QP kit. The remaining 14 samples were therefore extracted by the IHMS Protocol Q. All samples (*n* = 24) extracted by the IHMS Protocol Q were further subjected to shotgun metagenome sequencing and downstream analysis.

### Gut Microbial Composition Between HIV-1-ART Cases and HIV-1-Negative Controls

**FIGURE 3:**
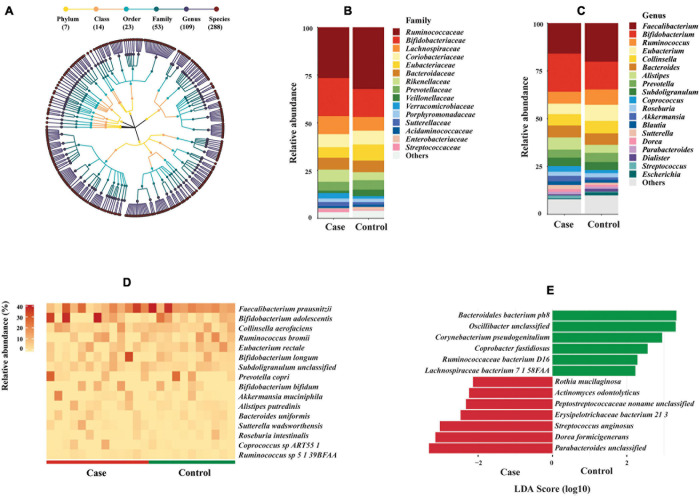
Bacterial composition and difference between HIV-1-antiretroviral therapy (ART) individuals (cases) and HIV-1-negative controls. **(A)** Taxonomic tree of bacterial taxa identified in this study. Each dot represents a taxonomic entity. From the inner to outer circles, the taxonomic levels range from phylum to species. Different colors of dots indicate different taxonomy levels according to the color key shown. Numbers in parentheses indicate the total number of unique taxonomies at each taxonomic level. **(B,C)** Barplots of main bacterial taxa at family and genus levels between cases and controls (average abundance >1% in either group). Main taxa at order level are shown in Supplementary Figure 2. **(D)** Heat map of abundant bacterial species (average abundance >1%) among individuals between cases and controls. The relative abundance of bacterial species is represented by a color gradient as indicated. The species were ordered by decreasing relative abundance. **(E)** Species biomarkers identified by linear discriminative analysis (LDA) effect size (LEfSe) analysis between cases (in red) and controls (in green). LDA scores (log 10) for the enriched species in controls are represented on the positive scale, while LDA-negative scores indicate enriched species in cases. The threshold used to consider a discriminative feature for the LDA score was set at >2. Taxonomic biomarkers at higher levels are shown in Supplementary Figure 2.

Surprisingly, the average proportion of filtered reads assigned to known bacterial species accounted for 39.9% of total reads, less than 0.1% of reads were assigned to archaea and virus, whereas 60% of reads could not be mapped to any known reference genome of different taxonomic groups by MetaPhlAn 2.0 (Supplementary Figure 1a and Supplementary Table S3). Given that the focus of this study was on bacterial communities, the downstream analysis was performed on bacteria only. Intriguingly, the bacterial species-level richness rapidly reached a plateau for all 24 samples at less than 5 million reads (Supplementary Figure 1b). MetaPhlAn 2.0 identified 288 bacterial species in the 24 fecal samples (Supplementary Table S4), belonging to 109 genera, 53 families, 23 orders, 14 classes, and seven phyla (Figure 3A). *Clostridiales* and *Bacteroidales* were the most abundant orders (Supplementary Figure 2a). *Ruminococcaceae* and *Bifidobacteriaceae* were the most abundant families; *Faecalibacterium* and *Bifidobacterium* were the most abundant genera (Figures 3B,C). The most abundant bacterial species observed in all fecal samples was *Faecalibacterium prausnitzii* (average relative abundance 15.97% in case group vs. 20.16% in control group), followed by *Bifidobacterium adolescentis* (11.17% case vs. 7.11% control), *Collinsella aerofaciens* (5.96% case vs. 6.51% control), and *Ruminococcus bromii* (4.16% case vs. 6.10% control). Sixteen species showed average relative abundance above 1% (Figure 3D). However, no significant difference of these abundant species was found between HIV-1-ART cases and HIV-1-negative controls.

Despite the small sample size, three families, five genera, and 13 species biomarkers were identified between the case and control groups (Figure 3E and Supplementary Figures 2b,c) using LEfSe biomarker discovery tool. Among the 13 species biomarkers, seven were identified as biomarkers for the case group and six for the control group (Figure 3E). Wilcoxon rank sum test was consistent with LDA with two exceptions (*Corynebacterium pseudogenitalium* and *Streptococcus anginosus*) (Supplementary Table S5A).

### The Altered Microbial Diversity in HIV-1-ART Cases Compared to Negative Controls

**FIGURE 4:**
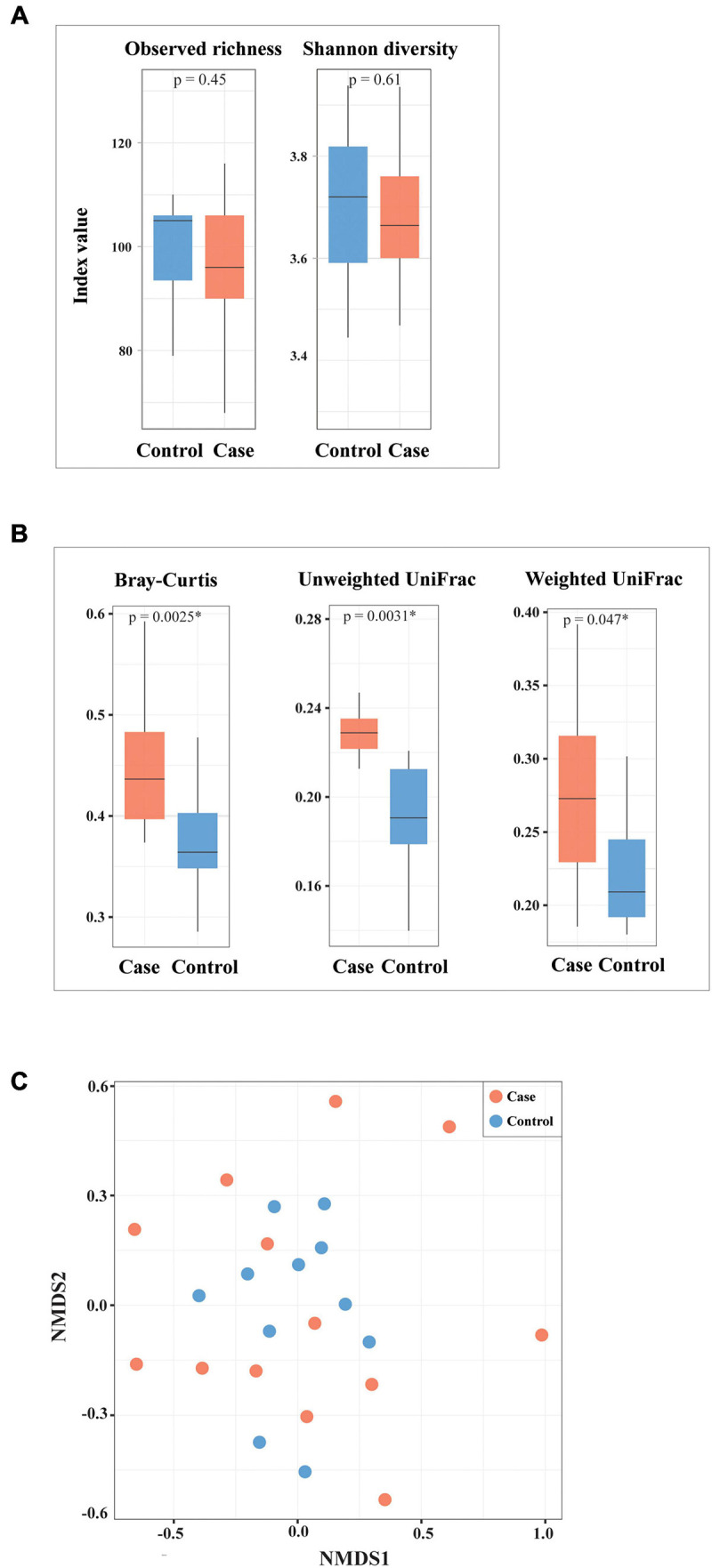
Alpha and beta diversity of bacterial species between cases and controls. **(A)** Alpha diversity assessed by observed species richness and Shannon diversity. **(B,C)** Beta diversity assessed with Bray–Curtis, weighted UniFrac, unweighted UniFrac dissimilarities, as well as non-metric multidimensional scaling (NMDS) based on Bray–Curtis distance. ^∗^Statistically significant.

A decreased tendency of microbial richness and Shannon diversity at species level was observed in HIV-1-ART cases compared to HIV-1-negative controls, though differences were not statistically significant (*p* > 0.05) (Figure 4A). Beta diversity assessed with Bray–Curtis, unweighted UniFrac, and weighted UniFrac dissimilarities was significantly higher in the case group compared to that in the control group (*p* = 0.0025, *p* = 0.0031, *p* = 0.047, respectively) (Figure 4B). NMDS with Bray–Curtis distance (Figure 4C) and UniFrac distances (data not shown) showed no significant separation between cases and controls, although samples from cases were more diversely distributed as compared to controls. Based on IGC analysis, no statistically significant difference was found in microbial gene richness between cases and controls.

### Microbial Functional Genes and Pathways in Cases and Controls

To assess the potential functionality of gut microbiota in HIV-1 infection, microbial genes were annotated and analyzed using different databases and functional systems: KEGG and MetaCyc pathways, KO. We found that 94.6% of filtered reads were unmapped or unintegrated against the KEGG database. The most abundant KEGG pathways identified in this study were biosynthesis of ansamycins (ko01051), valine, leucine and isoleucine biosynthesis (ko00290), ribosome (ko03010), D-Glutamine, and D-glutamate metabolism (ko00471) (Supplementary Figure 3a). Similarly, 95.4% of filtered reads were unmapped or unintegrated against the MetaCyc pathway database; the top MetaCyc pathways were adenosine ribonucleotide *de novo* biosynthesis (PWY-7219) (MetaCyc pathways class: nucleoside and nucleotide biosynthesis), UMP biosynthesis (PWY-5686) (nucleoside and nucleotide biosynthesis), UDP-N-acetylmuramoyl-pentapeptide biosynthesis II (lysine-containing) (PWY-6386) (cell structure biosynthesis), L-isoleucine biosynthesis I (from threonine) (ILEUSYN-PWY) (amino acid biosynthesis), pyruvate fermentation to isobutanol (PWY-7111) (generation of precursor metabolites and energy), and L-valine biosynthesis (VALSYN-PWY) (amino acid biosynthesis) (Supplementary Figure 3b). No statistically significant difference of these main pathways was found between the case and control groups. We found that one MetaCyc pathway, Bifidobacterium shunt (P124-PWY), also known as fructose-6-phosphate pathway, was statistically significantly enriched in the case group compared to the control group (Supplementary Table S5B). Additionally, the microbial communities in the case group were significantly enriched in genes encoding for enzymes such as xylulose-5-phosphate/fructose-6-phosphate phosphoketolase (K01621), which is key enzyme in fructose-6-phosphate pathway, as well as pyridoxamine 5’-phosphate oxidase (K00275), and dihydropteroate synthase (K00796) (Supplementary Table S5C).

### Association Between Gut Microbiome and CD4 + T-Cell Counts

**FIGURE 5:**
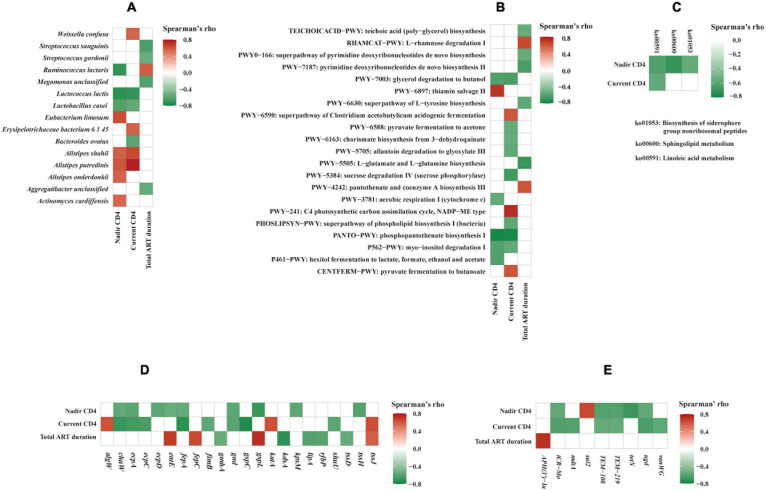
Correlation analysis of gut microbiome and clinical variables. Correlations between clinical variables and bacterial species **(A)**, MetaCyc pathways **(B)**, Kyoto Encyclopedia of Genes and Genomes (KEGG) pathways **(C)**, virulence factors genes **(D)**, and antimicrobial resistance genes **(E)**. Spearman’s correlation rho values are represented by color gradient as indicated (red is for positive, green is for negative correlation). Only statistically significant correlations (*p* < 0.05) are shown on the plots.

The relative abundance of bacterial species, functional genes, and pathways in relation to the CD4 + T-cell counts were analyzed by LDA, Wilcoxon rank sum test, as well as Spearman’s correlation analysis. LDA identified five species biomarkers (shown in Supplementary Figure 4 including *Lactococcus lactis*) for the lower CD4 + T-cell counts (LC) group and one (*Alistipes putredinis*) for the higher CD4 + T-cell counts (HC) group. Wilcoxon rank sum test was consistent with LDA with two exceptions (Supplementary Table S6A). Consistently, *A. putredinis* was positively correlated to both nadir CD4 + T-cell count (rho = 0.603, *p* = 0.0290) and current CD4 + T-cell count (rho = 0.779, *p* = 0.0017), while *Lactococcus lactis* was inversely correlated to nadir CD4 + T-cell count (rho = −0.733, *p* = 0.0044) and current CD4 + T-cell count (rho = −0.717, *p* = 0.0058) using Spearman’s correlation analysis (Figure 5A). The total ART duration was positively correlated to *Ruminococcus lactaris* (rho = 0.580, *p* = 0.0375) while inversely correlated to *Streptococcus sanguinis* (rho = −0.638, *p* = 0.0191), *Streptococcus gordonii* (rho = −0.554, *p* = 0.0497), *Megamonas* unclassified (rho = −0.620, *p* = 0.0237), and *Aggregatibacter* unclassified (rho = −0.559, *p* = 0.0470) (Figure 5A).

LDA identified one MetaCyc pathway biomarker, i.e., phosphopantothenate biosynthesis I (PANTO-PWY) among patients in the lower current CD4 + T-cell counts (LC) group compared to those with higher current CD4 + T-cell counts (HC) group (data not shown). Using Wilcoxon rank sum test, 22 additional MetaCyc pathways and one KEGG pathway chlorocyclohexane and chlorobenzene degradation (ko00361) were found to be significantly enriched in the LC group (Supplementary Tables S6B,C). At the gene family level, 57 KOs with characterized function were found to be significantly different between LC and HC groups (Supplementary Table S6D). The Spearman’s correlation analysis showed that three MetaCyc pathways were inversely correlated to both nadir and current CD4 + T-cell counts (Figure 5B). Of these, phosphopantothenate biosynthesis I (PANTO-PWY) showed strongly inverse correlations with both nadir CD4 + T-cell count (rho = −0.784, *p* = 0.0015) and current CD4 + T-cell count (rho = −0.780, *p* = 0.0017). Consistently, phosphopantothenate biosynthesis I (PANTO-PWY) and glycerol degradation to butanol (PWY-7003) pathways were significantly enriched in the LC group (*p* = 0.0012 and *p* = 0.0082, respectively) by Wilcoxon rank sum test (Supplementary Table S6B). These data suggested that MetaCyc pathways related to phosphopantothenate biosynthesis I and glycerol degradation to butanol were associated with low CD4 + T-cell count. We also observed correlations between three KEGG pathways and CD4 + T-cell counts (Figure 5C). For instance, linoleic acid metabolism pathway (ko00591) was inversely correlated to both nadir CD4 + T-cell count (rho = −0.630, *p* = 0.0211) and current CD4 + T-cell count (rho = −0.577, *p* = 0.0391), which was consistent with an earlier study (Vesterbacka et al., 2017).

### Difference of Bacterial Virulence Factors and Antimicrobial Resistance Genes Between Cases and Controls

We identified multiple bacterial virulence factors genes and AMR genes in the full dataset (Supplementary Tables S7, S8). It is noteworthy that more than 200 virulence factor genes were detected exclusively in HIV-1-ART cases, though their relative abundance was low. These genes encode critical bacterial virulence factors such as adhesion, toxin, hemolysin, type III secretion system, etc. (Supplementary Table S7). We found that 173 AMR genes were present in cases only; in addition, cases were significantly enriched in genes associated with tetracycline antibiotic resistance and antibiotic efflux pumps (*p* < 0.05) (Supplementary Table S8). Rarefaction analysis showed that sequencing depth critically affected the recovery of bacterial virulence factors genes and AMR genes, which was dramatically different from taxonomic profiling. A sequencing depth of 57 million reads per sample was insufficient to capture full spectrums of bacterial virulence factors genes and AMR genes in human fecal samples in this study (Supplementary Figures 5a,b).

We observed correlations between virulence factors genes, AMR genes, and clinical variables (Figures 5D,E). Both current and nadir CD4 + T-cell counts were correlated inversely to virulence genes encoding 6-phosphogluconate dehydrogenase (*gnd*), *E. coli* common pilus structural subunit EcpA (*ecpA*), ferrienterobactin outer membrane transporter (*fepA*), and oxygen-independent coproporphyrinogen III oxidase (*chuW*) (Figure 5D). While nadir CD4 + T-cell count was inversely correlated to several important virulence genes such as type VI secretion system ATPase TssH (*tssh*) (rho = −0.625, *p* = 0.0224), fimbria adhesin EcpD (*ecpD*) (rho = −0.596, *p* = 0.0317), type II secretion system protein L (*gspL*) (rho = −0.702, *p* = 0.0074), polysialic acid transport protein KpsM (*kpsM*) (rho = −0.588, *p* = 0.0346), and enterobactin synthase component E (*entE*) (rho = −0.556, *p* = 0.0483). Additionally, current CD4 + T-cell count was inversely correlated to genes encoding outer membrane usher protein EcpC (*ecpC*) (rho = −0.652, *p* = 0.0157), type II secretion system protein C (*gspC*) (rho = −0.765, *p* = 0.0023), permease of iron compound ABC transport system (*shuU*) (rho = −0.592, *p* = 0.0330), and type 1 fimbriae regulatory protein FimB (*fimB*) (rho = −0.584, *p* = 0.0362). Our study also indicated that HIV-1 infection is correlated to changes in AMR genes. Both current and nadir CD4 + T-cell counts were inversely correlated to genes associated with beta-lactam antibiotic resistance (*TEM*-108 and *TEM*-219) and peptide antibiotic resistance (*icr-Mo* and *ugd*) (Figure 5E). While nadir CD4 + T-cell count was inversely correlated to tetracycline antibiotic resistance gene *tetX* (rho = −0.694, p = 0.0085), current CD4 + T-cell count was inversely correlated to *mdtA* (rho = −0.624, *p* = 0.0226), which encodes for resistance-nodulation-cell division (RND) antibiotic efflux pump, and glycopeptide resistance gene *vanWG* (rho = −0.584, *p* = 0.0362). Total ART duration was strongly positively correlated to aminoglycoside antibiotic resistance gene *APH(3’)-Ia* (rho = 0.725, *p* = 0.0050).

## Discussion

In this study, we set up a standard shotgun metagenomics workflow through methodologies assessment including sample preparation in order to get a precise gut microbiome profile. We did a literature review and selected and evaluated two fecal DNA extraction protocols that performed better than other methods used in gut microbiome studies (Angebault et al., 2018; Lim et al., 2018; Fiedorova et al., 2019). We found the IHMS Protocol Q yielded significantly higher gDNA quantity and more stable gDNA quality than the commercial kit QP. It is possible that different gDNA quantity and quality may have an impact on the microbial structures and downstream analysis; further studies are warranted to investigate this. Notably, we found that only ∼40% of the total reads were assigned to known microbial taxa, whereas 60% of the reads could not be mapped to any known taxonomic group by MetaPhlAn 2.0. This was in line with previous studies estimating that approximately 40–60% of human gut microbes cannot be captured by current genome-based methods, despite the considerable efforts that have been made to culture and sequence members of the gut microbiome (Sunagawa et al., 2013; Nayfach et al., 2019). We assessed the shotgun metagenome sequencing depth required for characterizing human gut microbiome at microbial species and gene level. We found that less than 5 million reads per sample were sufficient to achieve almost a full bacterial composition, whereas an increasing number of microbial genes, including virulence factors genes and AMR genes, were still being recovered at a sequencing depth of 57 million reads. This finding is consistent with a recent metagenomics study performed on animal and environmental samples, indicating that taxonomic profiling is much more stable to sequencing depth than AMR gene content (Gweon et al., 2019). To the best of our knowledge, this is the first study depicting the shotgun metagenome sequencing depth requirement for simultaneously characterizing gut microbiome, virulome, and resistome in human fecal samples. Our findings will facilitate the selection of appropriate sequencing depth for human gut microbiome studies to obtain reliable results.

Accumulating studies, including ours, have shown that HIV-1 infection is associated with gut dysbiosis, reduced alpha diversity, and increased beta diversity of microbiota (Nowak et al., 2015; Kang and Cai, 2019; Flygel et al., 2020), which may persist after ART assumption (Vujkovic-Cvijin et al., 2013; Vazquez-Castellanos et al., 2015; Ji et al., 2018). Despite the study limits, e.g., small sample size and lack of treatment-naive HIV-1 patients, we observed similar microbiota alteration in HIV-1-ART individuals compared to negative controls. It should be noted that previous studies were mostly based on 16S rRNA gene sequencing, which can profile microbiota at the genus level. Our data showed similar changes of microbial diversity in HIV-1-infected individuals at the species level. We found that several bacterial species were significantly enriched in HIV-1-ART individuals. Three of these species, at their higher taxonomic level (i.e., *Peptostreptococcus*, *Erysipelotrichaceae*, and *Dorea*), have earlier been reported to be enriched in HIV-1-infected subjects (McHardy et al., 2013; Vujkovic-Cvijin et al., 2013; Dinh et al., 2015; Ling et al., 2016), indicating the potential role of these bacteria in HIV-1 pathogenesis.

To date, only a few studies have investigated functional genes and pathways in microbial communities and their roles in HIV-1 infection using whole metagenome sequencing (Vazquez-Castellanos et al., 2015; Lu et al., 2018; Guillen et al., 2019; Rocafort et al., 2019). None of these studies has assessed the proportion of functionally characterized genes in the samples. Remarkably, we found that more than 94% of filtered reads were unmapped or unintegrated against two established pathway databases, which was in line with a previous study using similar methods (Abu-Ali et al., 2018). These data suggest that most microbial genes in human feces remain functionally unknown. We found that “Bifidobacterium shunt,” also known as fructose-6-phosphate pathway, was significantly enriched in the case group. Consistently, gene encoding for the key enzyme in this pathway, i.e., xylulose-5-phosphate/fructose-6-phosphate phosphoketolase, which plays a key role in carbohydrate metabolism in a number of bacteria, was also enriched in the case group. This may indicate the potential role of the fructose-6-phosphate pathway in HIV-1 infection. Further larger studies with matched groups are essential to confirm this and unravel the potential underlying mechanisms.

We observed correlations between CD4 + T-cell count and gut microbiome in HIV-1-ART patients. For instance, both current and nadir CD4 + T-cell counts were inversely correlated to linoleic acid metabolism pathway, which is corroborated by previous findings that the fatty acid metabolism has an important role in the regulation of immune responses and immunological diseases (Yaqoob, 2003; Hosomi et al., 2020). Additionally, both current and nadir CD4 + T-cell counts were inversely correlated to pathways related to phosphopantothenate biosynthesis I, myo-inositol degradation I, glycerol degradation to butanol, implying that these pathways might play a role in the mechanism of immune cell regulation in HIV-1 infection. A previous study indicated that the microbial shift associated with immune deficiency in HIV-1 infection was related to increased bacterial virulence factors and AMR genes in the gut microbiome (Guillen et al., 2019). Consistently, we found important bacterial virulence factors such as adhesion, toxin, hemolysin, type III secretion system encoding genes that were exclusively present or enriched in the HIV-1-ART patients. AMR genes associated with tetracycline antibiotic resistance were significantly enriched in the case group. Several virulence factors genes and AMR genes were found to be correlated to CD4 + T-cell count and total ART duration. It is notable that the total ART duration was strongly positively correlated to abundance of aminoglycoside antibiotic resistance gene *APH(3’)-Ia*. We did not have access to earlier antibiotic treatment in HIV-1-ART individuals, but it is not unlikely that a more frequent use of antibiotics preceding the study in these patients could have contributed to these findings. Further studies are needed to validate the associations between HIV-1 infection, degree of immunodeficiency, and gut microbiome observed in this study.

We acknowledge limitations in this study. First, it is an exploratory microbiome study using deep shotgun metagenome sequencing on a small sample size, as its purpose was mainly to set up a standard whole metagenomics pipeline, thereby characterizing the gut microbiome profile associated with HIV-1 infection at the bacterial species and functional levels. Second, previous studies indicated that antiretroviral drugs themselves may impact the gut microbiota (Nowak et al., 2015; Ray et al., 2020). Since all HIV-1-infected participants in this study were on ART at the time of sampling, we could not differentiate the effect of ART and HIV-1 infection itself, respectively, on gut microbiome dysbiosis. Third, the associations between gut microbiome alteration and HIV-1 infection might be cofounded by factors such as gender, age, and sexual preference, which were not evaluated due to the small sample size. It should be noted that the gender difference in cases and controls might also be a confounder. In addition, it is difficult to identify microbial differences in composition and functions associated with disease status, as such differences can be partly due to genetic diversity among the human samples (Goodrich et al., 2014; Tierney et al., 2019). Furthermore, statistical correction for multiple testing was not applied because of the low sample size and the exploratory focus of the study. Therefore, further longitudinal studies on a larger scale of treatment-naive patients before and after ART, with matched groups by other confounders, are essential to validate our findings.

In conclusion, we set up whole metagenomics workflows and characterized gut microbiome in HIV-1-infected individuals on ART. The results indicate that gut dysbiosis in HIV-1 infection is potentially associated with changes in bacterial composition, functional genes and pathways, as well as gut microbial virulome and resistome. Further studies are essential to validate these findings and to evaluate potentially unmeasured confounders that might impact the gut microbiome. The methodological assessment shows that different DNA extraction protocols significantly affect the gDNA quantity and quality, and metagenome sequencing depth critically affects the recovery of microbial genes including bacterial virulence factors genes and AMR genes in human metagenomic samples. These findings will support the design of future human gut microbiome studies, particularly when virulome and resistome are of study interest.

## Data Availability Statement

The raw shotgun metagenome sequencing data and metadata from this study have been submitted to GenBank Sequence Read Archive under the BioProject ID PRJNA692830. All the codes are available at github: https://github.com/Asw614/Whole-genome-metagenomic-analysis-of-the-gut-microbiome.

## Ethics Statement

The studies involving human participants were reviewed and approved by The Regional Ethics Committee, Stockholm (2009/1485-31, 2013/1944-31/4, and 2014/920-3). The patients/participants provided their written informed consent to participate in this study.

## Author Contributions

AS and UN initiated the study and supervised the sample and data collection. XB performed the laboratory work, analyzed the data, and wrote the manuscript. AN performed the bioinformatics and statistical analyses with the supervision of UN and XB and contributed to the manuscript preparation. PN and SR contributed to the sample collection, data interpretation, and manuscript preparation. All authors reviewed, edited, and approved the final manuscript.

## Conflict of Interest

The authors declare that the research was conducted in the absence of any commercial or financial relationships that could be construed as a potential conflict of interest.

## Footnotes

1 http://www.microbiome-standards.org

2 https://www.bioinformatics.babraham.ac.uk/projects/trim_galore/
