# Supplementary material for: Whole-Genome Metagenomic Analysis of the Gut Microbiome in HIV-1-Infected Individuals on Antiretroviral Therapy
Source: Front Microbiol. 2021 Jun 25;12:667718. doi: 10.3389/fmicb.2021.667718 (PMC8267369; doi:10.3389/fmicb.2021.667718)
Supplement: Supplementary file 1 [file Data_Sheet_1.PDF]

Supplementary Materials

Table S1. Metagenome sequencing reads QC (.xlsx)

Table S2. Concentration and quality of extracted genomic DNA by two protocols (.xlsx)

Table S3. Composition of microbial communities in this study analyzed by MetaPhlAn2.0 (.xlsx)

Table S4. Bacterial species identified in this study and their relative abundance in each sample (.xlsx)

Table S5. Differential species and functional profiles identified by Wilcoxon rank sum test between cases and controls (.xlsx)

Table S6. Differential species and functional profiles identified by Wilcoxon rank sum test between higher and lower CD4+ T cell counts groups in HIV-1-ART cases (.xlsx)

Table S7. Bacterial virulence factor genes identified in this study (.xlsx)

Table S8. Antimicrobial resistance genes identified in this study (.xlsx)

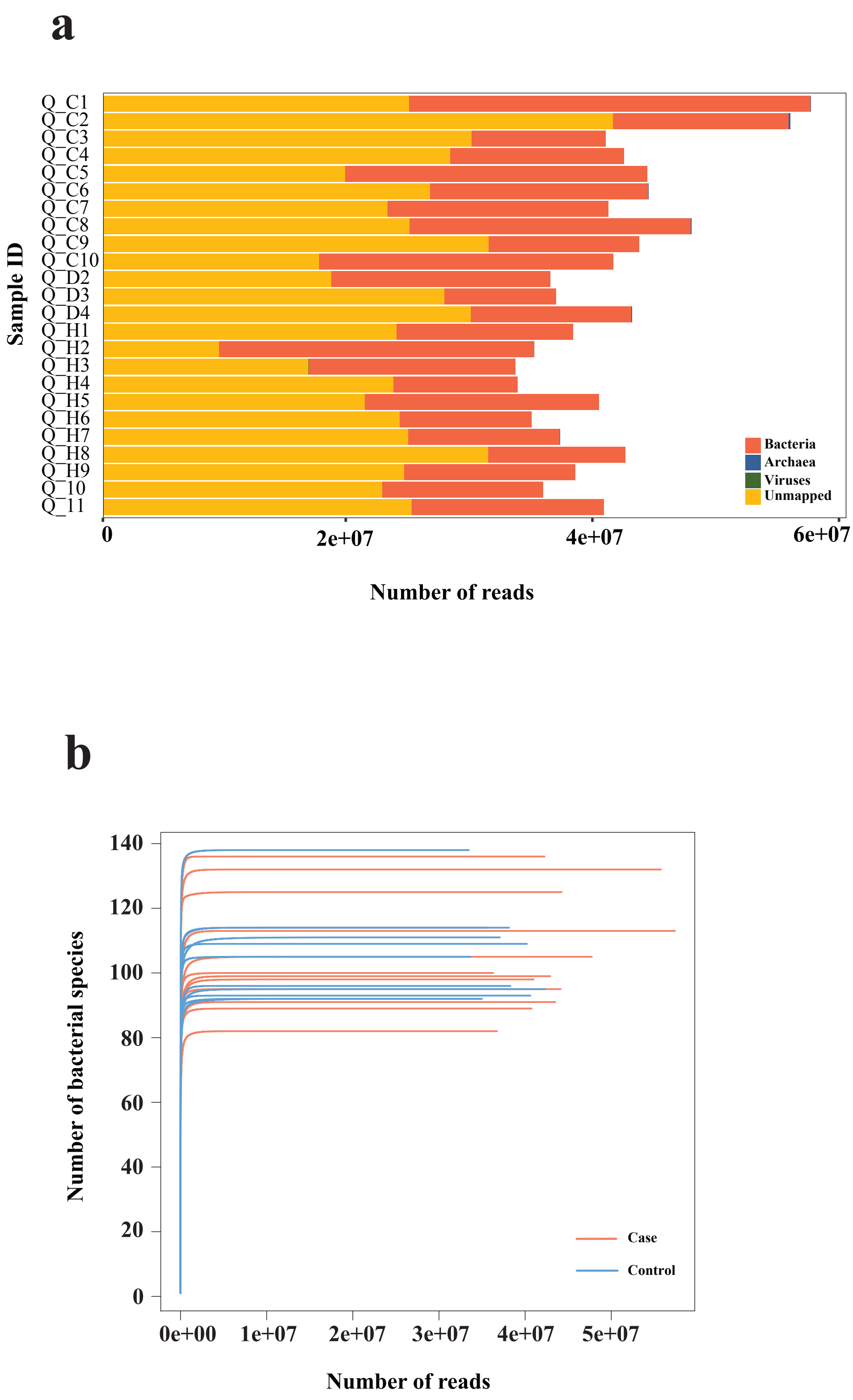

Figure S1. Summary of taxonomy profiling by MetaPhlAn 2.0. **a** Proportion of reads mapping to known reference genome sequences of different taxonomic groups by MetaPhlAn 2.0. Bacterial species accounted for 39.9% of total reads, archaea and viruses less than 0.1%, 60% were unmapped. **b** Rarefaction curves created for each sample to assess the saturation of different sequencing depth for recovery of bacterial species using MetaPhlAn 2.0.

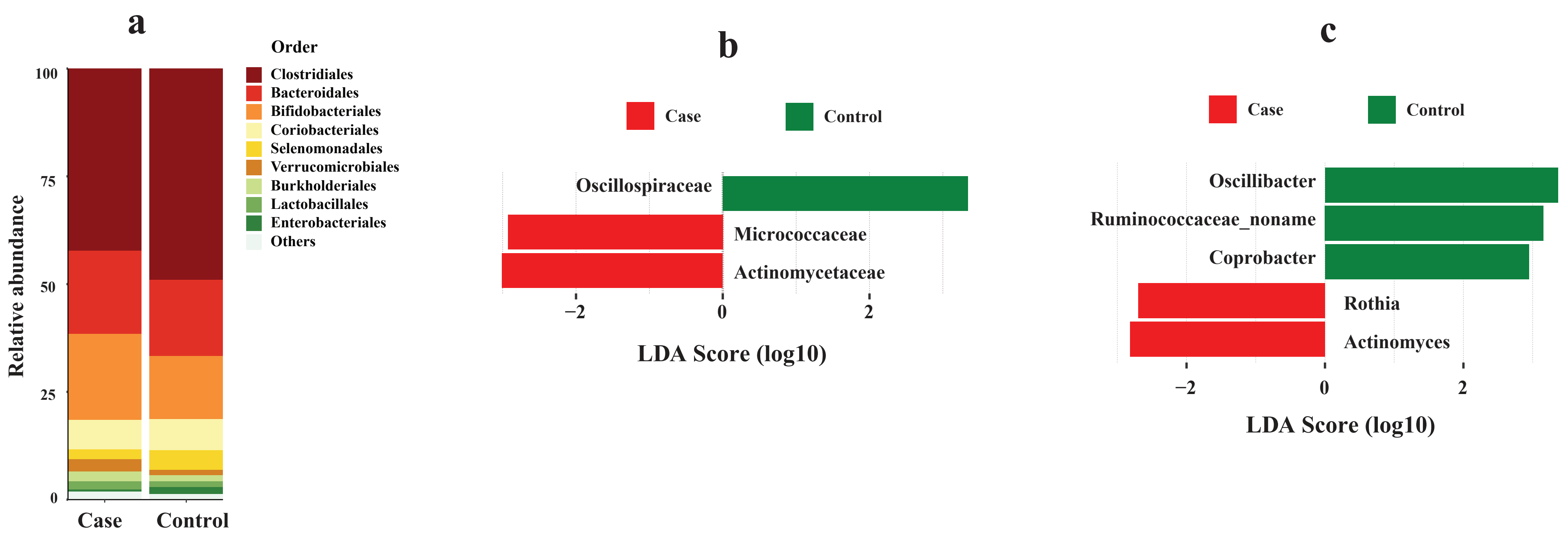

Figure S2. Bacterial composition and difference between HIV-1-ART individuals (cases) and HIV-1 negative controls at higher taxonomic level. **a** Barplot of main bacterial orders between cases and controls, orders with average relative abundance >1% in either group are shown. Main bacterial taxa at family, genus and species level are shown in Figure 3. **b** and **c** Taxonomic biomarkers at family (**b**) and genus (**c**) levels between cases and controls identified by linear discriminative analysis (LDA) effect size (LEfSe) analysis. LDA scores (log 10) for the enriched taxa in controls are represented on the positive scale (green), while LDA-negative scores indicate enriched taxa in cases (red). The threshold used to consider a discriminative feature for the LDA score was set at >2. No biomarker at order level was identified.

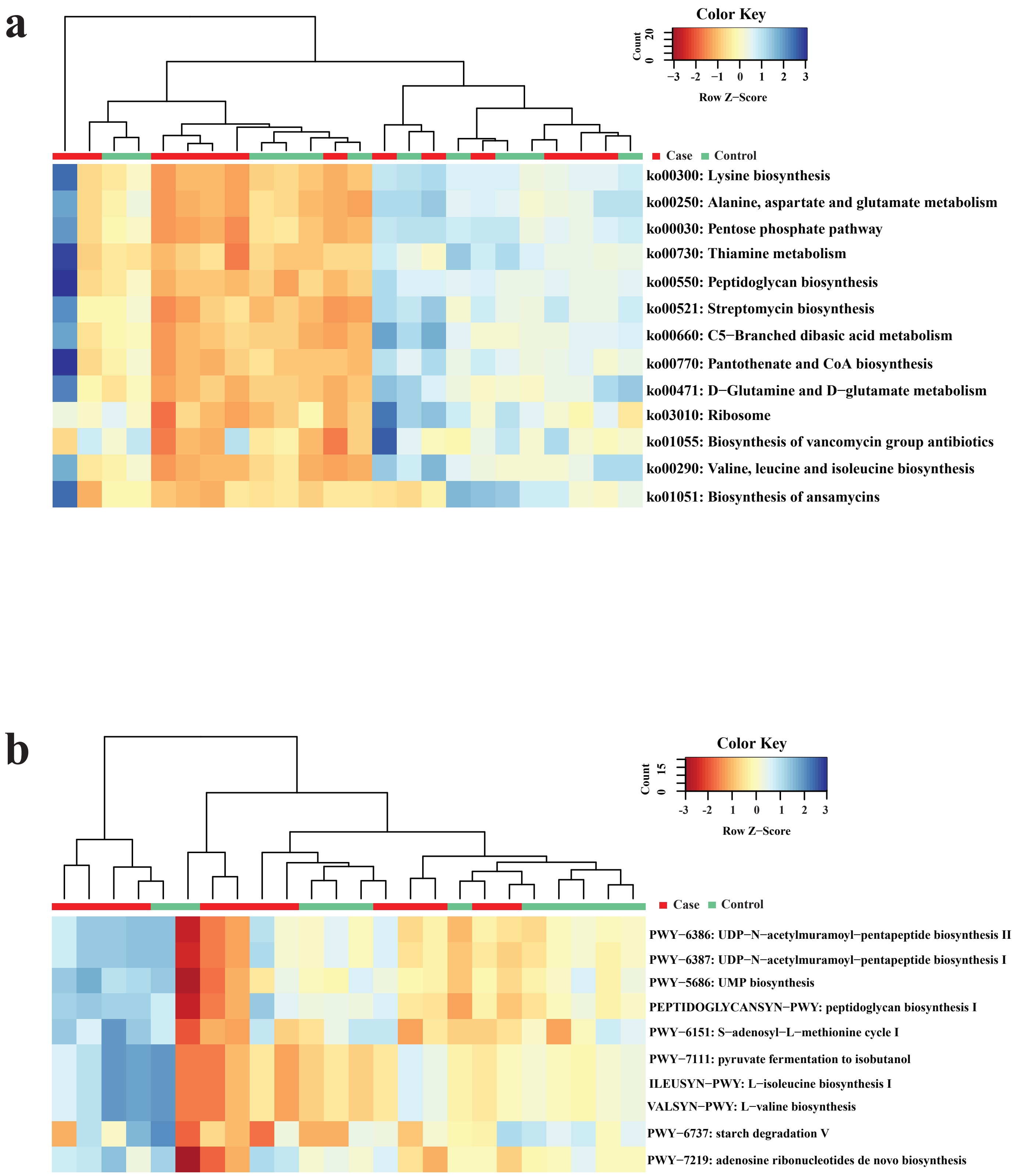

Figure S3. Heat map of main KEGG and MetaCyc pathways identified in this study. **a** Abundant KEGG pathways with average relative abundance > 0.1% in all samples. **b** Top ten MetaCyc pathways in all samples. No statistically significant difference was found for these main pathways between the case and control groups (p>0.05).

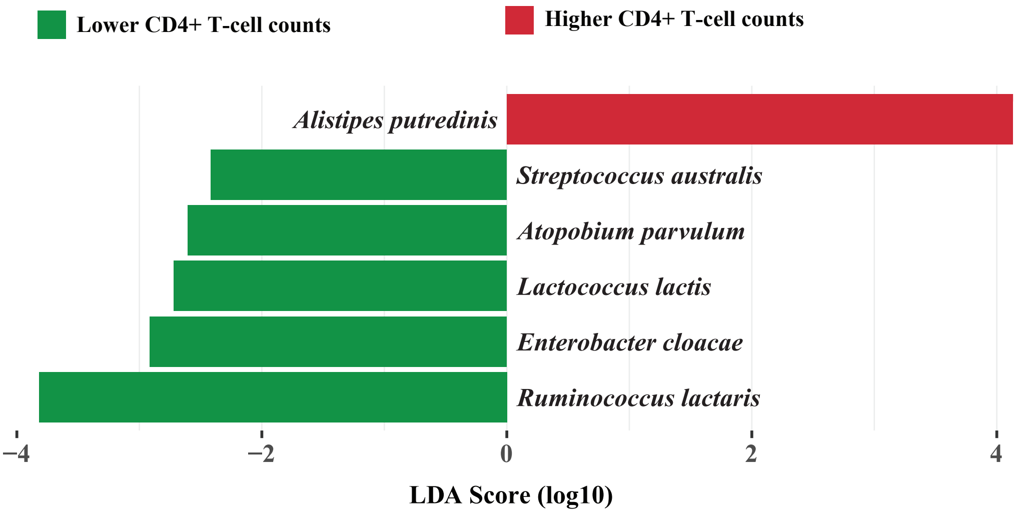

Figure S4. Linear discriminative analysis (LDA) effect size (LEfSe) analysis of bacterial species between higher CD4+ T-cell counts (HC) (≥740 cells/mm3) group and lower CD4+ T-cell counts (LC) (<740 cells/mm3) group. LDA scores (log 10) for enriched species in each group are represented on the positive scale and negative scale as indicated.

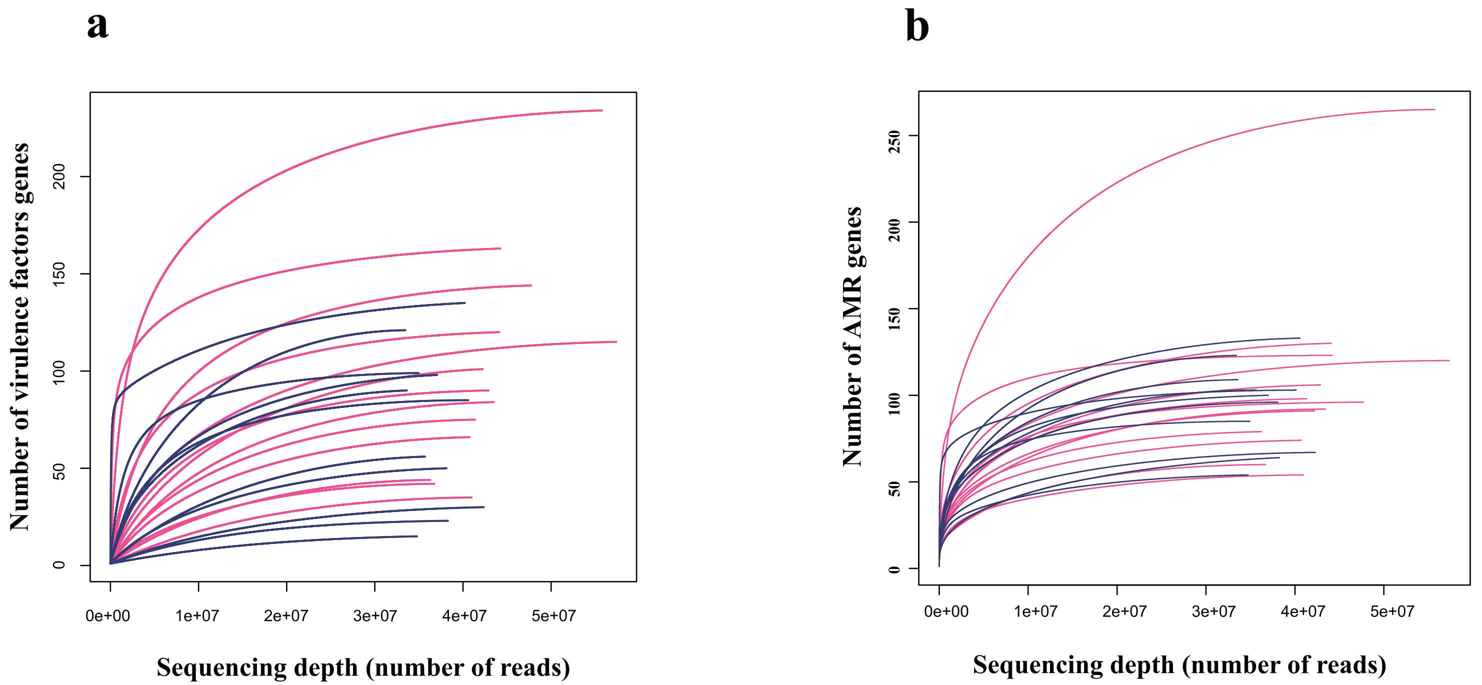

Figure S5. Rarefaction curves created for each sample to assess the saturation of samples at different sequencing depth for recovery bacterial virulence factors genes (**a**) and AMR genes (**b**).
